# *In Vivo*, *In Vitro*, and *In Silico* Characterization of Peptoids as Antimicrobial Agents

**DOI:** 10.1371/journal.pone.0135961

**Published:** 2016-02-05

**Authors:** Ann M. Czyzewski, Håvard Jenssen, Christopher D. Fjell, Matt Waldbrook, Nathaniel P. Chongsiriwatana, Eddie Yuen, Robert E. W. Hancock, Annelise E. Barron

**Affiliations:** 1 Department of Chemical and Biological Engineering, Northwestern University, 2145 Sheridan Road, E136, Evanston, IL, 60208, United States of America; 2 Centre for Microbial Diseases and Immunity Research, #232–2259 Lower Mall Research Station, University of British Columbia, Vancouver, BC V6T 1Z4, Canada; 3 Dept. of Science, Systems & Models, Roskilde University, Universitetsvej 1, DK-4000, Roskilde, Denmark; Purdue University, UNITED STATES

## Abstract

Bacterial resistance to conventional antibiotics is a global threat that has spurred the development of antimicrobial peptides (AMPs) and their mimetics as novel anti-infective agents. While the bioavailability of AMPs is often reduced due to protease activity, the non-natural structure of AMP mimetics renders them robust to proteolytic degradation, thus offering a distinct advantage for their clinical application. We explore the therapeutic potential of *N*-substituted glycines, or peptoids, as AMP mimics using a multi-faceted approach that includes *in silico*, *in vitro*, and *in vivo* techniques. We report a new QSAR model that we developed based on 27 diverse peptoid sequences, which accurately correlates antimicrobial peptoid structure with antimicrobial activity. We have identified a number of peptoids that have potent, broad-spectrum *in vitro* activity against multi-drug resistant bacterial strains. Lastly, using a murine model of invasive *S*. *aureus* infection, we demonstrate that one of the best candidate peptoids at 4 mg/kg significantly reduces with a two-log order the bacterial counts compared with saline-treated controls. Taken together, our results demonstrate the promising therapeutic potential of peptoids as antimicrobial agents.

## Introduction

Drug development in the golden age of antibiotics (the 1960s and 1970s) resulted in an unprecedented ability to control infections worldwide. However, initial successes bred a false sense of security that modern medicine could retain complete control over bacterial infections [1]. The emergence and re-emergence of multi-drug resistant (MDR) bacteria has since been recognized as an alarming threat to public health, and a dearth of novel antibiotic classes is creating significant unmet clinical need [2, 3]. Most new antibiotics are closely related in structure to existing ones, making the route to pathogen development of drug resistance short and inevitable [2]. The pharmaceutical industry’s waning interest in antibiotic development coupled with the inadequate management of existing drugs are additional factors contributing to the urgency of this global crisis [4, 5]. Among the most notable new and promising classes of broad spectrum antibiotic agents are the antimicrobial peptides (AMPs) and their mimics.

AMPs, also known as host defense peptides, are key components of innate immunity that have recently generated significant interest as innovative lead compounds for clinical use [6, 7]. While AMPs comprise a family of molecules diverse in source, length, and structure, these peptides are typically short (12 to 50 amino acids), carry a net positive charge (+ 2 to 9), and contain up to 50% hydrophobic amino acids [8]. These physicochemical properties permit interactions with microbial membranes and enable their typically broad-spectrum antimicrobial activity either by directly disrupting the membrane or permeabilizing it and acting on intracellular targets, ultimately causing cell death [9, 10]. Some cationic AMPs appear to interact with the polyanionic surface of lipopolysaccharide (LPS), destabilizing the outer membrane [11–13] before passing through by self-promoted uptake to the cytoplasmic membrane. It is unlikely that any class of antibiotic agents can entirely thwart the development of resistant bacterial strains [14–16], but it is clear that the non-receptor mediated and generalized mode of action employed by AMPs can be more robust to bacterial resistance than conventional antibiotics [10].

While the use of cationic antimicrobial peptides has met with some success [17], several recent clinical trials have failed to show efficacy for certain AMPs [10]. Statistically significant activity has been demonstrated for only one candidate peptide, MX-226, for treatment of topical infections. An issue limiting the applicability of AMP drugs is their susceptibility to protease activity, which can lead to an unfavorable pharmacokinetic profile and low bioavailability [10]. One approach to achieving selective, broad-spectrum antimicrobial activity through a generalized mode of action while avoiding protease susceptibility is the use of peptidomimetic, non-natural scaffolds [18, 19]. *N*-substituted glycines, or peptoids, are sequence-specific synthetic oligomers that can be designed to mimic the helical, cationic, and amphipathic structure of some AMPs. Certain peptoids have been shown to exhibit potent and selective antimicrobial activity and appear to employ mechanisms of action similar to those of their natural counterparts [20–24]. Peptoids are based on a backbone structure that is identical to that of peptides, however peptoid side chains are appended to the amide nitrogens rather than the α-carbons [25]. This modification has important structural implications. The achiral backbone structure of peptoids precludes backbone hydrogen bonding, but they can be induced to form stable polyproline type-I-like helices by the incorporation of bulky, α-chiral side chains [25–30].

Here we report a study that both extends the analogy between peptide and peptoid mechanisms of action, and demonstrates the proof-of-concept *in vivo* efficacy of peptoids against MDR pathogenic bacterial strains, with comparison to the activities of related peptides. The antimicrobial activities of peptoid-based AMP mimics is studied here using a multi-faceted approach that incorporates *in silico*, *in vitro*, and *in vivo* techniques. We have generated a library of short, helical peptoids that mimic classical amphipathic antimicrobial helical peptides, and incorporated selected previously reported peptoid sequences that have diverse structures, antimicrobial potencies, and cell selectivities [20, 21] into a QSAR (quantitative structure activity relationship) model using chemical descriptors [31]. As was previously shown for peptides [31], the QSAR model was able to accurately predict the minimum inhibitory concentration (MIC) of an excluded peptoid based on its structure. The *in vitro* antibacterial activity of a selected set of peptoids was evaluated against 20 pathogenic and multi-drug resistant bacterial strains in comparison to that of two clinically relevant antimicrobial peptides and four broad-spectrum, clinically utilized antibiotics. Several peptoids exhibited potency superior to that of comparator peptides and antibiotics against both Gram-negative and Gram positive-strains. An LPS binding assay was used to demonstrate that peptoid **1** was able to interact with the polyanionic component of Gram-negative bacteria to an extent similar to that of peptides. Lastly a proof-of-concept study using an invasive *Staphylococcus aureus* challenge model demonstrated the ability of peptoid **1** to achieve a statistically significant reduction of bacterial counts *in vivo* compared to a saline-treated control group.

## Materials and Methods

### Peptoid and peptide synthesis and purification

The peptoids were synthesized on an ABI 433 peptide synthesizer using the submonomer method [25] on Rink amide MBHA resin. Briefly, bromoacetic acid activated by diisopropylcarbodiimide is used to form a bromoacetylated intermediate on a terminal secondary amine group. Bromine is then substituted with the desired primary amine through an S_N_2 displacement, to build the peptoid chain. The synthesized peptoids were cleaved from the resin using trifluoroacetic-acid:triisopropylsilane:water (95:2.5:2.5, v:v:v) for 10 minutes. The peptoids were purified by reversed-phase HPLC using a C18 column and a linear acetonitrile/water (0.1% trifluoroacetic acid) gradient of 5%–95% acetonitrile over 45 minutes. Final purity was greater than 98% and correct molecular identity was verified using electrospray ion mass spectrometry. The control peptides were synthesized using solid phase Fmoc chemistry, purified to purity > 95% using reversed phase HPLC, and were analyzed by mass spectrometry by GenScript (Piscataway, NJ, USA).

### Antimicrobial testing against superbug strains

The MICs of test agents were measured using a modified broth micro-dilution method in Difco Mueller Hinton medium [32], on a panel of bacterial pathogens that were both susceptible and resistant to common antibiotics (S1 File). Briefly, serial dilutions were performed in 0.01% acetic acid containing 0.2% bovine serum albumin at 10-fold the desired final concentration. Ten μL of the 10-fold concentrated test reagents were added to each well of a 96-well polypropylene microtitre plate containing 90 μL of Mueller Hinton media per well. Bacteria were added to the plate from an overnight culture at a final concentration of 2–7 x 10^5^ CFU/mL and incubated overnight at 37°C. The MIC is defined as the concentration at which no growth was observed.

### QSAR modeling

QSAR descriptors were calculated using Molecular Operating Environment v2006.05 (Chemical Computation Group Inc., Montereal, Canada). A total of 233 descriptors were initially calculated based on a three-dimensional structure estimated from energy minimization using the generalized Born solvation model. A linear equation for predicting activity based on QSAR descriptors was constructed in two phases. First, a set of up to ten descriptors were chosen that gave the highest cross-validated regression between predicted and measured activity where activity was taken as the log10 of the MIC in molarity. Descriptors were considered in order of declining regression against activity (i.e. the first descriptor had the highest absolute correlation with activity; the next descriptor had the next highest). Initially, each descriptor was considered individually in a linear regression; the descriptor with the highest performance (described below) was selected. Next, the linear regression models were constructed using the previously identified descriptor with an additional term using each other descriptor. The model with highest performance with two descriptors was selected. This process was repeated for up to ten descriptors or until no improvements were found for additional descriptors.

Performance of the multiple regression models was calculated as the absolute correlation of the predicted to measured activity using a 10-fold cross-validation. For a 10-fold cross-validation, the data were randomly selected into ten sets. Nine of the ten sets were used to construct the multiple linear regression model; the model was then used to predict the activity of the set that was not used. By repeating the process for each set, predictions of activity were made for each peptoid without using that peptoid data itself in constructing the model.

In the second phase, a linear regression model was constructed for all peptoid data using only the descriptors identified. The standard errors and p-values of each parameter in the model were calculated. Where a p-value of a parameter was less than 0.1, the parameter was dropped and the model evaluated with the remaining descriptors. Parameters were dropped until all remaining descriptors had P-values > 0.1.

### Dansyl polymyxin B displacement assay

The Dansyl polymyxin B (DPX) displacement assay was carried out as described earlier [33, 34]. In brief, the fluorescence of DPX (Invitrogen) bound to LPS (*P*. *aeruginosa*) was measured by using a Luminescence spectrometer LS 50B (Perkin Elmer) with excitation and emission-wavelengths of 340 nm and 485 nm, respectively. A predetermined amount of DPX, resulting in 90% saturation of LPS, was added to 1 mL of 3 μg/mL of purified LPS. Small aliquots (5 μL of 1 μg/mL) of test compounds were added under constant stirring in the cuvette, and the displacement of DPX was measured for 30–60 seconds as a decrease in fluorescence. The process was repeated until maximum displacement was reached.

### Murine model of bacterial infection

Animal experiments were performed in accordance with University of British Columbia Care Ethics Committee approval and guidelines as per animal care certificate #A04-0020. Female CD1 mice (6–8 week old) were weighed, marked, and injected with 200 μL of *S*. *aureus* (ATCC 25923) ~10^9^ CFU/mouse suspended in Mueller Hinton broth containing 5% mucin and injected intraperitoneally. Four hours after infection, animals were treated with 4 mg/kg or 100μg per mouse of peptoid **1** (experimental group; n = 10) or an equivalent volume of saline (control group; n = 10). All animals were monitored two hours after each injection step, they were also evaluated at the end of the working day and the next morning a couple of hours prior to the experimental endpoint. Humane endpoints criteria; immobilization and shaking, were put down but none of the test subjects were evaluated to reach these endpoints prior to the experimental endpoint. The mice were euthanized 24 hours post-infection using CO_2_. The peritoneal cavity was exposed and washed with 5 mL PBS. The lavage was diluted to 10^−5^ in log order increments and spotted in duplicate onto Mueller Hinton agar plates. Plates were incubated overnight at 37°C and colonies were counted the following day. One animal in the saline treated control group was reported dead the morning after the infection, likely due to the infection load. This animal was assigned the highest colony forming unit count obtained in the experiment. The statistical differences between peptide treated and naïve mice were assessed using PRISM^®^ (GraphPad Software Inc., San Diego, CA) using contingency tables Chi-square test, with a confidence interval of 99%.

## Results

### QSAR model

**Fig 1:**
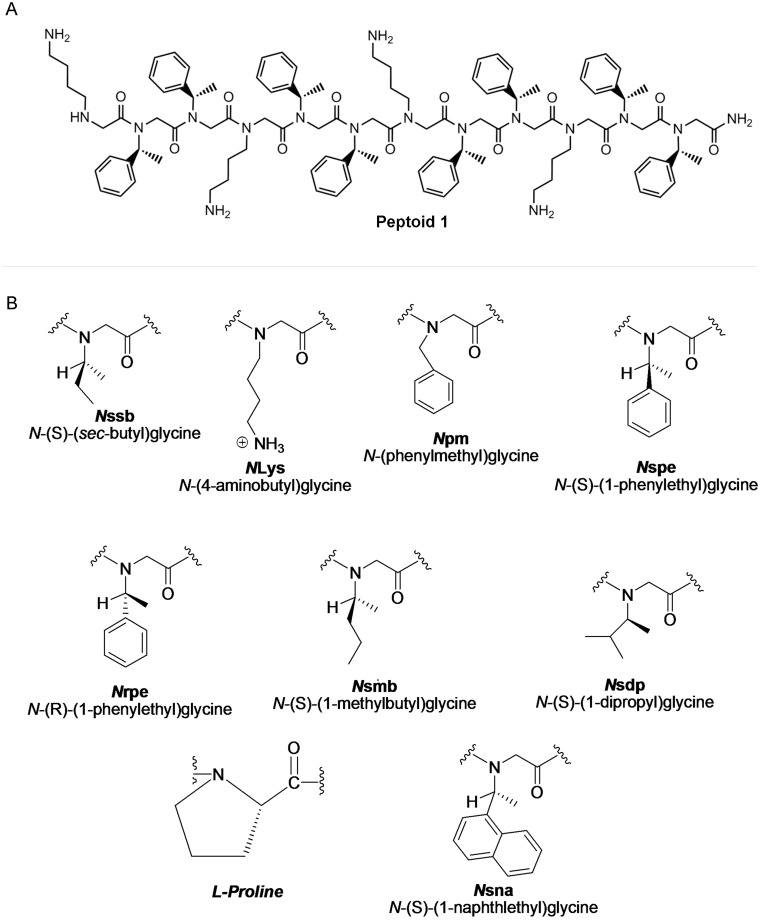
Chemical structures of (A) peptoid 1 and (B) the peptoid monomers.

We incorporated a select group of previously reported peptoid sequences with a broad range of physicochemical properties, antimicrobial potencies, and cell selectivities into a QSAR analysis capable of modeling the structural basis of peptoid antibacterial activity. All peptoid sequences were derived from the parent dodecamer, peptoid **1** (20), which is composed of 1/3 lysine-like, positively charged monomers (NLys) and 2/3 phenylalanine-like hydrophobic, aromatic monomers (Nspe) with the repeating sequence H-(NLys-Nspe-Nspe)_4_-NH (Fig 1A, Table 1). A computer simulation of the three-dimensional structure of peptoid **1** based on energy minimization (S1 File). All Peptoid sequences are summarized in Table 1 and the chemical structures of the side chains are shown in Fig 1B. Table 1 also summarizes the broad range of activities demonstrated by this library of compounds exhibited in the screening assays against *B*. *subtilis*, *E*. *coli*, erythrocytes (HD10/HD50), and NIH 3T3 cells. The peptoids demonstrate good selectivity for bacterial membranes over erytorcytes, however in parallel they do show an interesting and surprising inhibitory effect on the metabolism of the NIH 3T3 cells.

**Table 1:** Characteristics of antimicrobial peptoids, screened for activity against *B*. *subtilis* and *E*. *coli* bacterial strains.

Compound	MW	Sequence	*B*. *subtilis* (μM)	*E*. *coli* (μM)	HD_10_ / HD_50_	ID_50_ (μM)	HPLC elution (%ACN)	Ref.
Peptoid **1**	1819	H-(*N*Lys-*N*spe-*N*spe)_4_-NH_2_	1.6	6.3	21/100	5.1	65.1	[20, 21]
**1**_scr_	1819	H-(*N*spe)_2_-(*N*Lys-*N*spe)_3_-(*N*spe)_3_-*N*Lys-NH_2_	1.6	6.3	64/>200	8.5	61.1	
**1**-*N*Lys_5,11_	1753	H-(*N*Lys-*N*spe-*N*spe-*N*Lys-*N*Lys-*N*spe)_2_-NH_2_	0.78	50	>100 / >100	85	51.2	
**1**B-*N*Lys_4,10_	1753	H-(*N*spe-*N*Lys-*N*spe-*N*Lys-*N*Lys-*N*spe)_2_-NH_2_	0.78	50	>200 / >200	83	52.7	
**1**B_15mer_-*N*Lys_4,10_	2204	H-(*N*spe-*N*Lys-*N*spe-*N*Lys-*N*Lys-*N*spe)_2_-*N*spe-*N*Lys-*N*spe-NH_2_	0.78	50	>200 / >200	16	55.5	
**1**B_15mer_-*N*Lys_4,6,10_	2171	H-(*N*spe-*N*Lys)_2_-*N*Lys_2_-(*N*spe-*N*Lys)_2_-*N*Lys-*N*spe_2_-*N*Lys-Nspe-NH_2_	0.78	> 100	>200 / >200	40	50.8	
**1**_**6mer**_	918	H-(*N*Lys-*N*spe-*N*spe)_2_-NH_2_	> 100	> 100	ND	ND	41.0	[20, 21]
**1**_**9mer**_	1379	H-(*N*Lys-*N*spe-*N*spe)_3_-NH_2_	1.6	25	ND	ND	46.0	[20, 21]
**1**_**11mer**_	1658	H-(*N*Lys-*N*spe-*N*spe)_3_-*N*Lys-*N*spe-NH_2_	0.78	6.3	103 / >200	11	ND	
**1**-Pro_3_	1755	H-*N*Lys-*N*spe-L-Pro-(*N*Lys-*N*spe-*N*spe)_3_-NH_2_	1.6	12.5	74 / >200	12	63.0	
**1**-Pro_6_	1755	H-*N*Lys-*N*spe_2_-*N*Lys-*N*spe-L-Pro-(*N*Lys-*N*spe_2_)_2_-NH_2_	1.6	12.5	83 / >200	18	62.4	[20]
**1**-Pro_9_	1755	H-(*N*Lys-*N*spe_2_)_2_-*N*Lys-*N*spe-L-Pro-(*N*Lys-*N*spe_2_)-NH_2_	1.6	12.5	165 / >200	24	62.6	
**1-***N*rpe_3,6,9,12_	1819	H-(*N*Lys-*N*spe-*N*rpe)_4_-NH_2_	1.6	6.3	16 / 67	3.8	63.5	
**1**_ach_	1701	H-(*N*Lys-*N*pm-*N*pm)_4_-NH_2_	1.6	12.5	183 / >200	16	59.8	
**1**_ach_-*N*spe_2_	1721	H-(*N*Lys-*Ns*pe-*N*pm)- (*N*Lys-*N*pm-*N*pm)_3_-NH_2_	0.78	6.3	160 / >200	11	60.8	
**1**_ach_-*N*spe_12_	1721	H-(*N*Lys-*N*pm-*N*pm)_3_-(*N*Lys-*N*pm-*N*spe)-NH_2_	1.6	6.3	164 / >200	15	62.0	
**1**-*N*pm_2,3,8,9_	1763	H-(*N*Lys-*N*pm-*N*pm-*N*Lys-*N*spe-*N*spe)_2_-NH_2_	1.6	6.3	39 / >200	15	63.3	
**1**-*N*pm_2,5,8,11_	1763	H-(*N*Lys-*N*pm-*N*spe)_4_-NH_2_	1.6	6.3	87 / >200	6.8	63.6	
**1**-*N*sdp_all_	1547	H-(*N*Lys-*N*sdp-*N*sdp)_4_-NH_2_	0.78	25	>200 / >200	64	63.2	
**1**-*N*sdp_2,3,8,9_	1683	H-(*N*Lys-*N*sdp-*N*sdp-*N*Lys-*N*spe-*N*spe)_2_-NH_2_	0.78	12.5	77 / >200	19	64.7	
**1**-*N*sdp_2.5.8.11_	1683	H-(*N*Lys-*N*sdp-*N*spe)_4_-NH_2_	0.78	12.5	111 / >200	20	63.8	
**1-***N*sna_6,12_	1919	H-(*N*Lys-*Ns*pe-*N*spe-*N*Lys-*N*spe-*N*sna)_2_-NH_2_	1.6	50	ND	ND	53.0	[20, 21]

Minimal inhibitory concentration test of *B*. *subtilis* ATCC 6633 and *E*. *coli* ATCC 35218. HD10/HD50 representing the hemolytic activity of the tested peptoids. The dose found to inhibit the metabolic activity of NIH 3T3 cells using the colorimetric tetrazolium salt based MTS assay is reported as the ID_50_ (inhibitory dose). HPLC elution is reported as the average percentage of acetonitrile (ACN) in the solvent mixture upon compound elution for three injections. A linear acetonitrile/water (0.1% trifluoroacetic acid) gradient of 5%–95% acetonitrile over 45 minutes was run on a C18 column. Note: ND signifies not determined.

The model was built based on antibacterial activity measurements against only *E*. *coli* (ATCC 35218), excluding only the parent sequence, peptoid **1**. Against *E*. *coli*, these sequences ranged from potent (MIC ~ 6.3 uM) to inactive (> 100 uM). The three-dimensional structures of the peptoids were projected, and a total of 233 descriptors that were available in MOE (Molecular Operating Environment) were calculated. In addition, we created descriptors that were the products of these 233 MOE descriptors, resulting in a set of 27,494 descriptors. Of these, the majority did not vary between the peptoids or were highly correlated (Pearson correlation >0.95 or <-0.95) and thus eliminated, resulting in a total of 916 descriptors for modeling.

**Fig 2:**
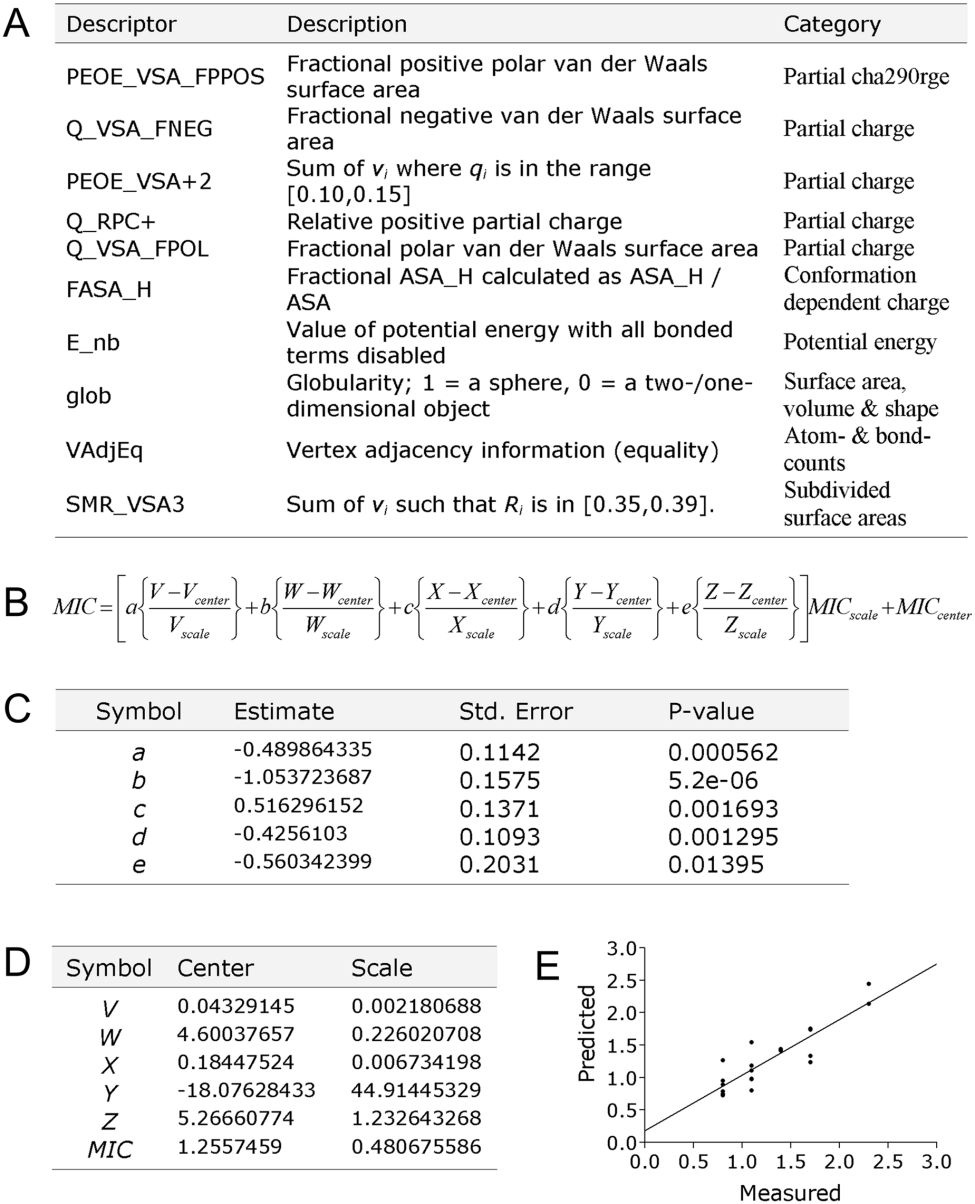
Model for predicting peptoid antimicrobial activity against Gram-negative *E*. *coli*. **(a)** gives an overview of the ten descriptors contributing the most to the predictive model. **(b)** gives the equation for calculating the predicted MIC (log10[MIC molar]) activity for any given peptoid, using the products of the descriptors in given as *V*, *W*, *X*, *Y* and *Z*, being (PEOE_VSA_FPPOS x Q_VSA_FNEG), (PEOE_VSA_plus2 x Q_RPC_plus), (Q_VSA_FPOL x FASA_H), (E_nb x glob) and (VAdjEq x SMR_VSA3), respectively (calculated separately for the specific peptoid). **(c)** The equation uses factors *a*, *b*, *c*, *d* and *e*, estimated contribution for each of the descriptor product elements, the accuracy and preciseness for *a*, *b*, *c*, *d* and *e* are indicated with standard error and p-values, and **(d)** the constant factors for centering and scaling of *V*, *W*, *X*, *Y*, *Z* and the *MIC* activities (forming the basis for the predictive model). **(e)** Illustrates predicted and measured are log(MIC in molar) for the peptoids in the generated QSAR solution, using a 10x cross-validated model (R_CV_ = 0,8892 and R_CV_^2^ = 0,7907).

The most important descriptors for explaining activity were identified using multiple linear regression models. Ten descriptors were identified that yielded the highest regression in a 10-fold cross-validation, in which 90% of the data was modeled and used to predict the activities of the remaining 10% a total of 10 times. These descriptors were then combined to construct a multiple linear regression model, shown in Fig 2. The activity of the excluded peptoid **1** was then predicted (calculated) to be 7.0 μM against *E*. *coli*, using this QSAR model based on the equations illustrated in Fig 2. This predicted activity is remarkably similar to the measured value (6.3 μM), demonstrating the accuracy of the QSAR model and creating a solid foundation for the future optimization and design of peptoid-based AMP mimics.

### Broad-spectrum antibacterial peptoid activity

The antimicrobial activities of a subset of peptoids with varying characteristics and activities were further tested against 20 multi-drug resistant (MDR), clinically relevant pathogens (Tables 2 and 3, S1 File). MSI-78 [35] and MX-226 [36], two widely studied and clinically-investigated AMPs, as well as four commonly used antibiotics (the aminoglycoside tobramycin, the fluoroquinolone ciprofloxacin, the carbapenem β-lactam imipenem, and the cephalosporin ceftazidime) [31] were included in this study to provide a basis of comparison to other classes of antimicrobial agents. MX-226, also known as Omiganan^®^ (Migenix, Inc., Vancouver, British Columbia, Canada), is an indolicidin derivative that is currently being developed to reduce the incidence of device-related infections. In Phase III clinical trails, the topical application of MX-226 pentahydrocloride in a 1% gel (Omigard) led to a significant, 21% reduction of colonization of central venous catheters, and a 50% reduction in tunnel infections (www.migenix.com/prod_226.html) [36, 37]. MSI-78 (pexiganan) is a 22-amino acid analogue of the peptide magainin-2, which was first developed by Magainin Pharmaceuticals Inc. (now Genaera; http://www.genaera.com), and was clinically tested for efficacy in the treatment of diabetic foot ulcers. Phase III clinical trials showed that MSI-78 eliminated or significantly reduced infection in over 90% of patients while exhibiting a favorable toxicity profile, yet FDA approval was denied in 1999 because improved efficacy above standard treatment could not be demonstrated [37].

**Table 2:** The activities of selected peptoids against Gram-negative multi-drug-resistant “Superbugs”, compared to some of the most highly utilized antibiotics; aminoglycoside tobramycin, the fluoroquinolone ciprofloxacin, the carbapenem imipenem and the cephalosporin ceftazidime, in addition to MX-226 [31] and MSI-78. ESBL = Extended-spectrum β-lactamase producing organism; MDR = multi-drug resistant organism.

Bacterium	Peptides, peptoids and antibiotics MIC (μg/mL)																								
	#1	#2	#3	#4	#5	#6	#7	#7	#9	#10	#11	#12	#13	#14	#15	#16	#17	#18	#19	#20	#21	#22	#23	#24	#25
***P*. *aeruginosa***																									
H103 (wild type)	1	32	2	16	2	4	4	1	8	8	8	8	4	4	4	4	4	32	8	8	4	0.5	0.1	2	2
9 (MDR)	16	128	16	256	64	128	128	32	128	256	256	32	64	64	64	64	64	256	256	256	128	>128	128	128	>128
198 (MDR)	4	256	4	32	8	32	16	16	16	16	32	8	8	8	8	8	8	128	32	32	16	>128	32	32	128
213 (MDR)	8	64	4	16	8	16	4	8	8	16	16	8	8	8	4	4	4	64	8	16	8	>128	64	128	>128
LES400 (MDR)	4	128	2	16	8	16	8	16	8	8	16	4	4	4	4	4	8	32	8	16	8	4	0.5	1	32
H1027 (MDR)	2	64	2	4	0.13	0.25	0.5	0.13	2	4	2	4	2	2	1	2	2	4	2	4	2	32	1	8	128
H1030 (MDR)	8	128	4	64	64	256	8	64	16	16	32	8	64	32	32	16	16	32	8	16	32	8	0.3	8	32
***P*. *maltophilia***																									
ATCC13637	2	32	2	2	0.5	0.25	0.25	0.5	1	2	2	2	4	2	1	1	2	1	1	1	1	4	0.3	128	0.3
***E*. *cloacae***																									
218R Class C β-lactamase	2	16	2	32	32	32	16	32	16	16	32	4	16	16	16	8	8	32	8	16	32	0.5	0.1	0.1	32
***E*. *coli***																									
63103 (ESBL)	16	64	2	64	128	128	256	256	16	16	32	4	16	16	16	8	8	64	16	16	16	64	>128	0.1	128
64771 (ESBL)	2	64	2	16	32	32	64	128	8	8	8	4	8	8	4	4	4	16	8	8	8	128	>128	0.1	>128
***K*. *pneumonia***																									
61962 (ESBL)	32	256	4	128	256	256	256	256	32	64	128	8	128	64	128	32	32	128	32	64	64	32	0.1	0.1	>128
63575 (ESBL)	8	128	4	64	128	128	32	128	32	32	64	16	64	32	32	16	16	128	32	32	32	16	64	0.1	>128

The table gives MIC values measured in 3–5 replicates for a spectrum of multi-resistant Superbugs with appropriate wild type control strains. The MIC values are given as (μg/mL) though the peptides roughly are 6–8 times larger than the conventional drugs. Peptides, peptoids and antibiotics numbering; #1 MSI-78, #2 MX-226, #3 Peptoid **1**, #4 **1**_scr_, #5 **1**-*N*Lys_5,11_, #6 **1**B-*N*Lys_4,10_, #7 **1**B_15mer_-*N*Lys_4,10_, #8 **1**B_15mer_-*N*Lys_4,6,10_, #9 **1**-Pro_3_, #10 **1**-Pro_6_, #11 **1**-Pro_9_, #12 **1**-*N*rpe_3,6,9,12_, #13 **1**_achiral_, #14 **1**_ach_-*N*spe_2_, #15 **1**_ach_-*N*spe_12_, #16 **1**-*N*pm_2,3,8,9_, #17 **1**-*N*pm_2,5,8,11_, #18 **1**-*N*sdp_all_, #19 **1**-*N*sdp_2,3,8,9_, #20 **1**-*N*sdp_2,5,8,11_, #21 **2**-*N*sna_6,12_, #22 Tobramycin, #23 Ciprofloxacin, #24 Imipenem and #25 Ceftazidime.

**Table 3:** The activities of selected peptoids against Gram-positive multi-drug-resistant “Superbugs”, compared to some of the most highly utilized antibiotics; aminoglycoside tobramycin, the fluoroquinolone ciprofloxacin, the carbapenem imipenem and the cephalosporin ceftazidime, in addition to MX-226 [31] and MSI-78. MRSA = Methicillin resistant *S*. *aureus*; VRE = Vancomycin resistant *Enterococcus*.

Bacterium	Peptides, peptoids and antibiotics MIC (μg/mL)																								
	#1	#2	#3	#4	#5	#6	#7	#7	#9	#10	#11	#12	#13	#14	#15	#16	#17	#18	#19	#20	#21	#22	#23	#24	#25
***S*. *aureus***																									
ATCC25923	64	16	2	8	64	256	32	256	4	4	8	4	4	4	4	2	4	16	4	8	4	0.3	0.1	0.1	16
C623 (MRSA)	16	32	2	8	64	256	16	128	4	8	8	4	4	4	8	2	8	8	4	8	4	>128	2	0.1	64
***E*. *faecalis***																									
ATCC29212	16	128	2	8	256	256	128	256	8	8	16	4	8	16	8	4	4	64	4	8	4	16	0.3	0.5	128
W61950 (VRE)	256	256	8	32	256	256	128	128	16	32	32	8	32	32	16	8	16	128	16	32	16	>128	32	2	>128
F43559 (VRE)	16	256	4	8	256	256	256	256	8	8	8	4	32	16	16	8	4	128	16	32	8	32	32	2	>128
***E*. *faecium***																									
mic80 (VRE)	4	64	2	8	16	32	32	32	4	8	8	4	4	4	4	4	4	16	4	4	4	128	32	128	>128
T62764 (VRE)	8	128	2	2	8	16	4	4	2	1	2	1	2	2	1	1	1	4	1	2	2	>128	128	>128	>128

The table gives MIC values measured in 3–5 replicates for a spectrum of multi-resistant Superbugs with appropriate wild type control strains. The MIC values are given as (μg/mL) though the peptides roughly are 6–8 times larger than the conventional drugs. Peptides, peptoids and antibiotics numbering; #1 MSI-78, #2 MX-226, #3 Peptoid **1**, #4 **1**_scr_, #5 **1**-*N*Lys_5,11_, #6 **1**B-*N*Lys_4,10_, #7 **1**B_15mer_-*N*Lys_4,10_, #8 **1**B_15mer_-*N*Lys_4,6,10_, #9 **1**-Pro_3_, #10 **1**-Pro_6_, #11 **1**-Pro_9_, #12 **1**-*N*rpe_3,6,9,12_, #13 **1**_achiral_, #14 **1**_ach_-*N*spe_2_, #15 **1**_ach_-*N*spe_12_, #16 **1**-*N*pm_2,3,8,9_, #17 **1**-*N*pm_2,5,8,11_, #18 **1**-*N*sdp_all_, #19 **1**-*N*sdp_2,3,8,9_, #20 **1**-*N*sdp_2,5,8,11_, #21 **2**-*N*sna_6,12_, #22 Tobramycin, #23 Ciprofloxacin, #24 Imipenem and #25 Ceftazidime.

Out of the 19 tested peptoids, 14 compounds demonstrated extremely broad-spectrum activity. In general, the activity against multi-drug resistant strains was highly correlated with those observed in the preliminary screen against *E*. *coli*; only certain clinically isolated *Pseudomonas* strains appeared to be intrinsically resistant to most of the tested peptoids. Peptoid **1** was found to be highly potent against 19 of the 20 bacterial strains tested and compared favorably to that of MX-226; the MICs of peptoid **1** ranged from 2 to 16 μg/mL, while those of MX-226 ranged from 16 to 256 μg/mL. Interestingly, the isolate that was most resistant to peptoid **1** was an MDR *P*. *aeruginosa* that was also polymyxin B resistant (MIC 64 μg/mL) due to overexpression of the PhoPQ and PmrAB 2-component regulators and downstream *arn* LPS-modification operon [38]. The potency of MSI-78 was approximately equal to that of peptoid **1** against *P*. *aeruginosa*, *P*. *maltophilia*, and *E*. *cloacae*; however, peptoid **1** demonstrated superior potency against two Gram-negative isolates and all Gram-positive strains, *S*. *aureus*, *E*. *faecalis*, and *E*. *faecium*. Against all Gram-positive strains tested, the MICs of peptoid **1** ranged from 2–8 μg/mL (median of 2 μg/mL), while those of MSI-78 ranged from 4–256 μg/mL (median of 16 μg/mL). These results suggest that peptoid **1** could be a promising candidate to treat some of the most recalcitrant and dangerous human infections.

### LPS binding activity of peptoid 1

We measured the LPS binding activity of peptoid **1** and comparator AMPs in order to investigate the nature of the interactions of these compounds with the outer membrane of Gram-negative species. The outer membrane of Gram-negative bacteria not only serves as a physical barrier to external stresses, but also provides structural integrity and plays a major role in the host’s immune response as a major antigen and the primary intrinsic (endo)toxin of Gram-negative bacteria, contributing to sepsis [11]. LPS is a polyanionic glycolipid, and is the major lipid component found within the outer surface layer of the Gram-negative bacterial outer membrane. LPS has divalent cationic binding sites that are stabilized and partially neutralized by divalent cations such as Mg^2+^ and Ca^2+^; such interactions contribute to outer membrane stability. Through the process of self-promoted uptake [39, 40], cationic peptides are able to displace divalent cations, disrupting LPS cross-bridging and destabilizing the outer membrane, in a process that promotes enhanced influx of the peptides.

The fluorescently labeled lipopeptide dansyl polymyxin B (DPX) has been shown to bind strongly to LPS, and this binding result in an enhanced fluorescence of the dansyl group [33]. The ability of other cationic molecules to displace DPX molecules bound to LPS (monitored by reduction in fluorescence) assesses their relative ability to bind to LPS [33, 34]. While the highly cationic MSI-78 (+10 charge) displaced 100% of bound DPX (equivalent to that of polymyxin B), peptoid **1** and MX-226 displaced 71% and 73% of bound DPX, respectively. These results correlate well with previous work, which demonstrated that a variety of polycations were able to displace between 63% and 100% of bound DPX [33]. A second parameter characterizing the LPS/polycation interaction is the I_50_, which is defined as the concentration of a polycation that displaces 50% of LPS-bound DPX. The I_50_ is inversely related to the relative affinity of each molecule for binding sites on LPS. For these compounds, I_50_ results indicated the order of decreasing LPS affinity as follows: MSI-78 (I_50_ = 1.4 μM) > Peptoid **1** (I_50_ = 2.6 μM) > polymyxin B (I_50_ = 3.2 μM) > MX-226 (I_50_ = 5.0 μM).

### *In vivo* biocompatibility and *S*. *aureus* clearance of peptoid 1

This dosage was selected to be on the conservative end of the dosage range typically evaluated for AMPs (1 mg/kg to 24 mg/kg) in other animal studies [35, 41, 42]. To screen for acute signs of toxicity, three healthy mice were injected intraperitoneally with 4 mg/kg (100 μg per mouse) of peptoid **1**, and a second control group with an equivalent volume of saline. At the conclusion of the 24 hours post-injection observation period, all mice appeared to be healthy and exhibited normal activity and no weight loss or abject morbidity (decreased movement, abnormal gait, piloerection, isolation in cage, or hunched abdomen).

**Fig 3:**
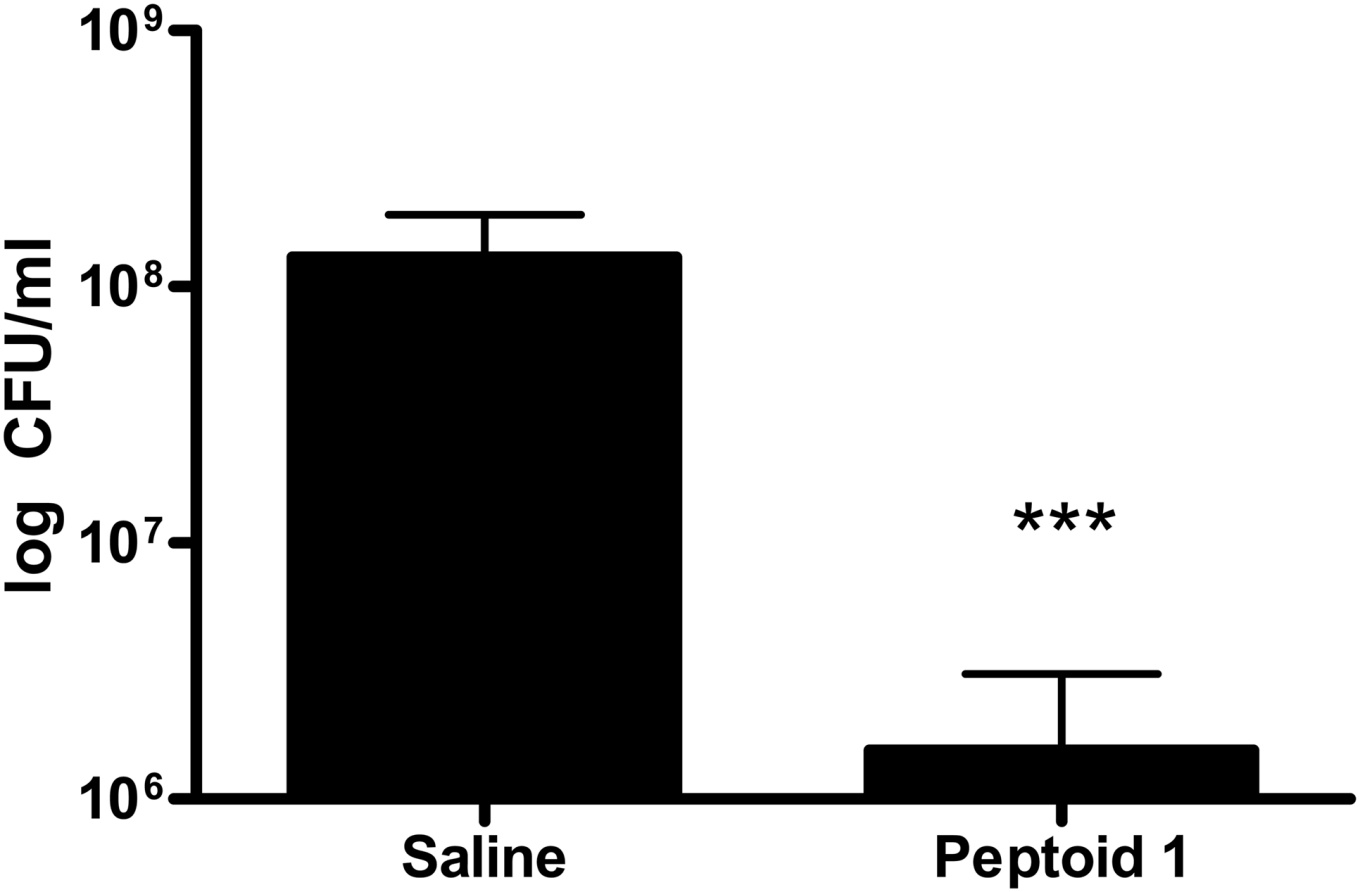
*In vivo* efficacy of peptoid 1. Four hours after intraperitoneal i.p. challenge with methicillin susceptible *S*. *aureus*, peptoid **1** was administered locally at a concentration of 4 mg/kg. Colony forming units (CFU) in the peritoneal lavage fluid from individual mice (plated in duplicate) at 24 hours are shown in the vehicle (saline) and peptoid 1 treatment groups. The graph indicates the geometric mean of each group. Dead animals at 24 hours were assigned the highest colony count observed in the experiment. *** indicates P<0.0001 by contingency Chi-square analysis, with a confidence interval of 99%.

We utilized an established murine model of invasive bacterial infection [41] to investigate the *in vivo* efficacy of peptoid **1** to treat an infection with *S*. *aureus*, a leading cause of nosocomial infections. The data in Fig 3 shows that bacterial counts in the peritoneal lavage fluid were significantly reduced (p < 0.0001) in mice treated with peptoid **1**, compared to saline-treated controls.

## Discussion

We leveraged the breadth in activities demonstrated by these peptoids to build a QSAR model capable of modeling the structural basis of peptoid antimicrobial activity. Much work has been invested in developing robust QSAR solutions for predicting the antimicrobial activities of synthetic AMPs [43, 44]. The transfer of experience and knowledge from this work has allowed us to build a fairly precise model, which appears to accurately predict peptoid antibacterial activity, based on the analysis of a set of structurally diverse peptoids (Fig 1). Investigating the nature of the ten chemical descriptors used in this presented model, it is apparent that the six most influential descriptors all are directly linked to peptoid charge (partial charge or conformational dependent charge) (Fig 2a). These are all sophisticated charge measurements interpreting the charge distribution over the entire chemical structure, and not just a simple net charge estimate summarizing the number of charge side chains. This is in good accordance with observation from other structure activity studies on AMP [31, 44–46], indicating that the antibacterial mechanism of action of AMPs and peptoids might be founded on the same principles. The four last descriptors in the model are related to molecular shape, surface area, and potential energy, suggesting that peptoid antimicrobial activity is also affected by molecular size. These results demonstrate the impact that computational techniques can have on potentially streamlining the rational design of potent antimicrobial peptoids in the future.

A subset of the peptoid library was tested side-by-side with two comparator antimicrobial peptides, for their activities against a panel of the most pathogenic, multi-drug resistant strains, including both Gram-negative (13 strains) and Gram-positive (7 strains) species. The MICs determined for antimicrobial peptides MX-226 and MSI-78 were consistent with previously reported values for these compounds against a similar panel of species [35, 37]. The broad-spectrum activity profile of peptoid **1** was found to be superior to those of the other peptoids, and to both MSI-78 and MX-226. The MIC of peptoid **1** ≤ 8 μg/mL for 19 out of the 20 pathogens tested; this was the case for only 12 of 20 organisms for MSI-78 and none of 20 for MX-226. Interestingly, MSI-78 and peptoid **1** showed similar activities against most Gram-negative strains, but MSI-78 was found to have reduced activity against most of the Gram-positive strains (MRSA and VRE). The MICs of peptoid **1** (2–16 μg/mL) were generally about 10-fold lower than those of MX-226 (16–256 μg/mL), demonstrating the superior *in vitro* antimicrobial activity of peptoid **1**. While it has been shown that *in vitro* activity and *in vivo* efficacy are not always directly correlated [41, 47], the potent, broad-spectrum activity of peptoid **1** against multi-drug-resistant bacterial cultures is a very encouraging result.

LPS affinity was studied to understand in greater detail the probable initial interactions of peptoid **1** with the outer surface of Gram-negative bacteria. The reduced competitiveness of peptoid **1** and MX-226 for sites on LPS (71% and 73% displacement, respectively) compared to that of MSI-78 and polymyxin B (100% displacement) suggests that all sites are not equally accessible to all cationic molecules [33]. Several properties of the cationic molecules that may influence their binding affinities for diverse sites on LPS include molecular size, net charge, hydrophobicity, and overall steric bulk, as well as specific molecular structure. The relative competitiveness of these molecules, however, does not translate directly into their relative affinity for LPS, a property that is inversely related to the I_50_. While both MSI-78 (I_50_ = 1.4 μM) and peptoid **1** (I_50_ = 2.6 μM) had stronger affinity for LPS than polymyxin B (I_50_ = 3.2 μM), MX-226 (I_50_ = 5.0 μM) demonstrated a weaker affinity. All of the cationic molecules tested have greater affinity for LPS than the most common cell envelope divalent cation, Mg^2+^ (620 μM) [48], suggesting that they can all initially displace Mg^2+^ and bind to the outer membrane. Interestingly, peptoid **1** and MSI-78 had the best activity against Gram-negative bacteria (Table 2) and also the highest LPS binding affinity. This correlation is reasonable, since the LPS binding affinity of other polycations correlates well with their ability to permeabilize outer membranes [33].

While LPS binding affinity is not necessarily synonymous with endotoxin neutralizing activity [49, 50], the strong LPS binding of peptoid **1** is also a promising result that encourages further studies. LPS is one of the primary causes of sepsis, a serious condition that affects many hospital patients [35]. Gram-negative bacteria in septic patients release LPS, which initiates a cascade of pro-inflammatory events. Many endotoxemia treatments have been investigated, including polymyxin B and a variety of cationic peptides [51]. In a clinical study, polymyxin B immobilized on a surface was shown to improve the survival rates of moderately septic patients, but was ineffective in patients with severe sepsis [52]. MSI-78 was found to not only reduce endotoxin plasma levels in a rat model of sepsis, but also to reduce bacterial counts by four orders of magnitude using the caecal ligation and puncture model of bowel perforation and sepsis [35]. The strong LPS binding affinity of peptoid **1** suggests that it could be a candidate anti-sepsis molecule.

Lastly, we studied the ability of peptoid **1** to treat an infection *in vivo* using a murine model of invasive *S*. *aureus* bacterial challenge. At a concentration of 4 mg/kg, peptoid **1** apparently caused no medium-term toxicity; treatment at this concentration resulted in an average two-log order reduction in bacterial counts in the peritonium. Moreover, mortality was reduced by 75% in the peptoid **1**-treated group compared to saline-treated controls. While other groups have published *in vivo* results with several types of non-natural AMP mimetics [42, 53, 54], this is the first report of bacterial count reduction after treatment with a helical peptoid-based AMP mimetic *in vivo*. This encouraging proof-of-concept result with peptoid **1** could usher in a new era in the development of peptoids as a class of clinically useful antimicrobial agents. Several lines of study that warrant further investigation include (1) optimal peptoid design for *in vivo* efficacy and safety; (2) optimal dosage and full toxicity profile; (3) the efficacy of peptoid treatment compared to AMP treatment; (4) the ability to treat a polyclonal infection using an animal sepsis model [35], and (5) the metabolic fate of peptoid-based antimicrobial agents.

In summary, this study was designed to combine the power of *in silico*, *in vitro*, and *in vivo* techniques to probe the therapeutic potential of peptoids as a new class of antimicrobial agents. The QSAR modeling results not only underscore the analogous behavior of peptoids and AMPs, but also demonstrate the utility of computer models to facilitate the design of future generations of peptoids. *In vitro* results suggested that peptoid **1** has potent antimicrobial activities against a range of Gram-positive and Gram-negative strains, which is apparently superior to those of comparator AMPs and overall better against Superbug organisms than four of the most highly used conventional antibiotics in our society. The relatively strong affinity of peptoid **1** for anionic binding sites on LPS suggest that it can displace stabilizing divalent cations and permeabilize the outer membrane of Gram-negative bacteria through self-promoted uptake. Most importantly, we present evidence that peptoid **1** can reduce colony forming units and mortality compared to a saline-treated control group in a murine model of invasive *S*. *aureus* challenge. Taken together, these results underscore the promising therapeutic potential of peptoids as a new class of clinically useful antimicrobial agents.

## Supporting Information

S1 File (DOC)
