# Supplementary material for: In Vivo, In Vitro, and In Silico Characterization of Peptoids as Antimicrobial Agents
Source: PLoS One. 2016 Feb 5;11(2):e0135961. doi: 10.1371/journal.pone.0135961 (PMC4744035; doi:10.1371/journal.pone.0135961)
Supplement: S1 File — (DOC) [file pone.0135961.s001.doc]

**Supporting Information (S1 File)**

***In vivo*, *in vitro*, and *in silico* characterization of peptoids as antimicrobial agents**

**Ann M. Czyzewski, Håvard Jenssen, Christopher D. Fjell, Matt Waldbrook, Nathaniel P. Chongsiriwatana, Eddie Yuen, Robert E.W. Hancock, Annelise E. Barron**

# Matherials and Methods

## Antimicrobial activity screening assay

MICs against *B. subtilis* and *E. coli* screening bacterial strains were determined using serial dilution in 96-well microtiter plates in accordance with CLSI M7-A6 protocols. Briefly, peptoid solution in cation-adjusted Mueller-Hinton broth was prepared by 2:1 serial dilution with a total volume of 50 µL per well. In experimental wells, 50 µL of bacterial inoculums (1 x 10^6^ CFU/mL) were added to the peptoid solutions. Positive controls consisted of 50 µL inoculums added to 50 µL cation-adjusted Mueller-Hinton broth containing no peptoid, while negative controls contained only cation-adjusted Mueller-Hinton broth. The MIC was reported as the lowest concentration of peptoid that completely inhibited bacterial growth after incubation at 35 ^o^C for 16 hours. Experiments were reproducible among three independent replicates, each consisting of two parallel trials.

Screening of peptoid antimicrobial activity against a panel of multidrug resistant superbugs was done according to the protocol described in the manuscript. The bacterial strains used in this panel included; *P.* *aeruginosa* PAO1 strain H103 [1], *P. maltophilia* ATCC#13637, *S. aureus* ATCC#25923 [1], *E. faecalis* ATCC#29212 [2], and *E. cloacae* 218R, constitutively expressing Class C chromosomal β-lactamase [3], were from the Hancock lab strains collection. An MRSA clinical isolate was kindly provided by Anthony Chow (Vancouver General Hospital, Vancouver, Canada). Two *Klebsiella pneumoniae* and two *E. coli* clinical isolates expressing extended spectrum β-lactamases (ESBL) were kindly provided by George Zhanel (Health Sciences Centre, Winnipeg, Canada). Vancomycin-resistant clinical isolates of *Enterococcus faecalis* and *E. faecium* were obtained from Ana M. Paccagnella (BC Centre for Disease Control, Vancouver, Canada). Three clinical isolates (#9, #198 and #213) of multi-drug resistant *P.* *aeruginosa*, were kindly provided by Carlos Kiffer (University of São Paulo, Brazil). These isolates all have resistance to piperacillin/tazobactam, meropenem, ceftazidime, ciprofloxacin and cefepime, and while #9 is also polymyxin B resistant. Three *P.* *aeruginosa* clinical isolates of the Liverpool epidemic strain (LES) (H1027, H1030 and LES400) [4] were all kindly provided by Craig Winstanley (University of Liverpool, UK). LES400 was resistant to gentamicin and tobramicin, while H1030 showed resistance to colistin, amikacin, gentamicin and tobramicin. All tested bacterial strains were categorized as biohazard level 2 pathogens.

## Metabolic activity, MTS assays

NIH 3T3 mouse fibroblast cells were cultured in complete Dulbecco’s Modified Eagle’s Media. A peptoid solution plate (100 µL per well) was prepared by serial dilution of aqueous peptoid stock solutions in Hank’s balanced salt solution. A day-old cell monolayer was washed thoroughly with Hank’s balanced salt solution and replenished with 100 µL Hank’s balanced salt solution per well. Peptoid solutions were transferred onto the day-old cell monolayers, which contained approximately 5000 cells per well. MTS reagent (40 uL per well) was added to each well, and the plate was incubated at 37 ^o^C for 3 hours, after which absorbance at 490 nm was determined using a plate reader. Percent inhibition was defined as [1 – (A – A_test blank_) / (A_control_ – A_blank_)] x 100, where A is the absorbance of the test well and A_control_ the average absorbance of wells with cells exposed to media and MTS (no peptoid). A_test blank_ (media, MTS, and peptoid) and A_blank_ (media and MTS) were background absorbances measured in the absence of cells. The average of six replicate trials is reported.

# Results

**
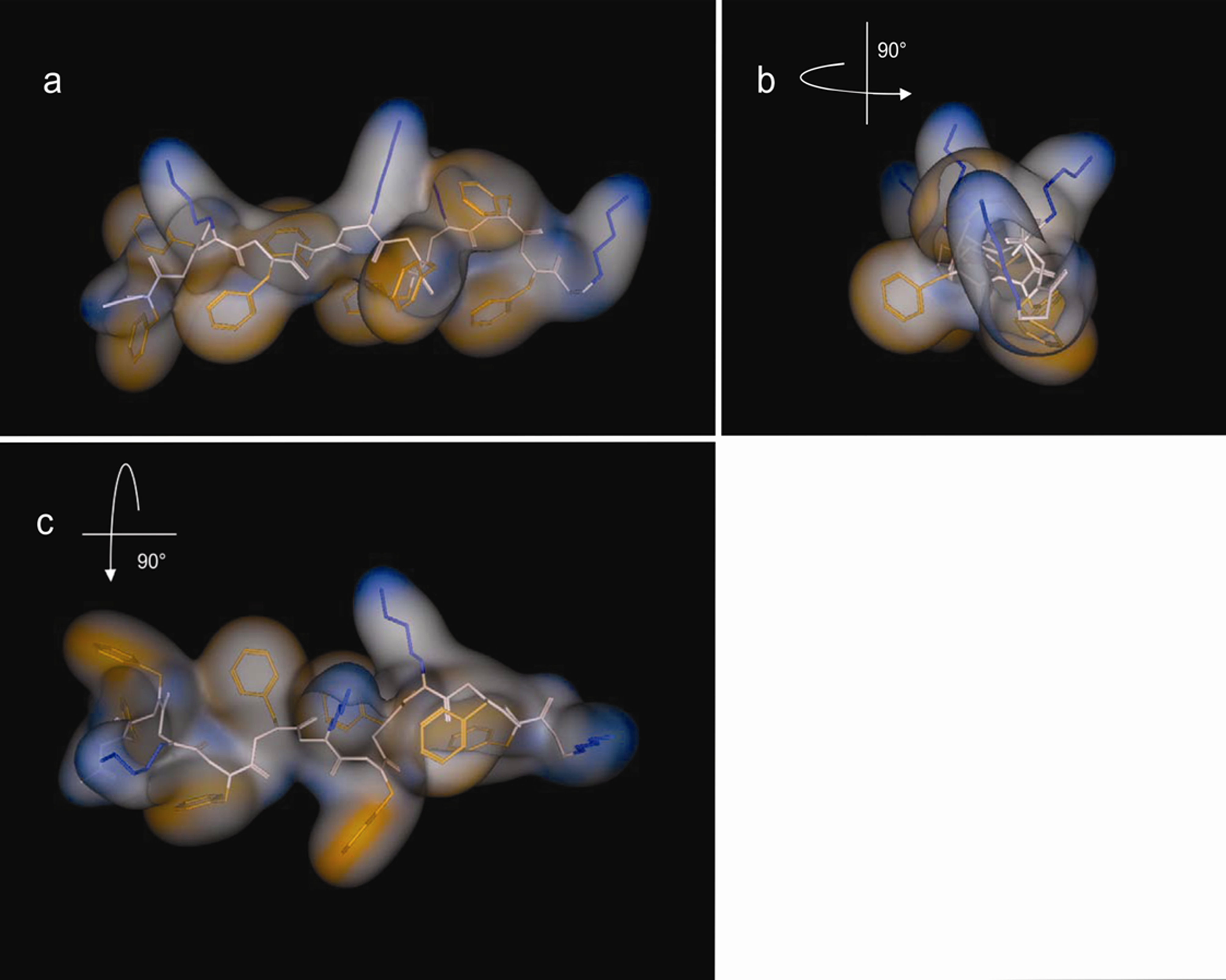
**

**S1 Fig. Computer-simulated three-dimensional structure of peptoid 1**. The structural projection has been generated using MOE with all-atom Merck molecular force-field (MMFF94x) and the compatible generalized Born solvation model considering partial charge distribution and energy minimization. (**a**) The simulation illustrates an overall helical structural with the four charged *N*Lys [(N-4-aminobutyl) glycine] residues (side chain colored blue) situated on one side of the molecule. To better illustrate the special positioning of the charge residues diagram **a** has been rotated (**b**) 90° around the *Y*-axis and (**c**) 90° around the *X*-axis. This structural projection is more precise and somewhat more complex than illustrated earlier [5].

# References

1. Wu M, Hancock REW (1999) Improved derivatives of bactenecin, a cyclic dodecameric antimicrobial cationic peptide. Antimicrob Agents Chemother 43: 1274-1276.

2. Hilpert K, Volkmer-Engert R, Walter T, Hancock REW (2005) High-throughput generation of small antibacterial peptides with improved activity. Nat Biotechnol 23: 1008-1012.

3. Bellido F, Pechere JC, Hancock REW (1991) Reevaluation of the factors involved in the efficacy of new beta-lactams against Enterobacter cloacae. Antimicrob Agents Chemother 35: 73-78.

4. Salunkhe, P. Smart CH, Morgan JA, Panagea S, Walshaw MJ, Hart CA, et al. (2005) A cystic fibrosis epidemic strain of Pseudomonas aeruginosa displays enhanced virulence and antimicrobial resistance. J Bacteriol 187, 4908-20.

5. Chongsiriwatana NP, Patch JA, Czyzewski AM, Dohm MT, Ivankin A, Gidalevitz D, et al. (2008) Peptoids that mimic the structure, function, and mechanism of helical antimicrobial peptides. Proc Natl Acad Sci U S A 105: 2794-2799.
